# Phylogenetic inference under the balanced minimum evolution criterion via semidefinite programming

**DOI:** 10.1093/bioinformatics/btag591

**Published:** 2026-08-06

**Authors:** Pavel Skums

**Affiliations:** School of Computing, University of Connecticut, Storrs, CT 06269, United States

## Abstract

**Motivation:**

In this study, we investigate the application of Semidefinite Programming (SDP) to phylogenetics. SDP is a powerful optimization framework that seeks to optimize a linear objective function over the cone of positive semidefinite matrices. As a convex optimization problem, SDP generalizes linear programming and provides relaxations for many combinatorial optimization problems. However, despite its many applications, SDP remains largely unused in computational biology.

**Results:**

We show how SDP relaxations can be designed and used for phylogenetic inference. We consider the Balanced Minimum Evolution (BME) problem, a widely used model in distance-based phylogenetics, and introduce an algorithm that combines an SDP relaxation with a rounding scheme that iteratively converts relaxed solutions into valid tree topologies. Experiments on simulated and empirical datasets show that the method enables accurate phylogenetic reconstruction.

**Availability and Implementation:**

The code and data are available at https://github.com/compbel/SDPTree (DOI 10.5281/zenodo.20838318).

## 1 Introduction

Phylogenetics plays a fundamental role in modeling evolutionary relationships and population dynamics across a wide range of domains, including molecular epidemiology, cancer genomics, developmental biology and genomic anthropology. A common feature of almost all phylogenetic is that the corresponding optimization problems are NP-hard (Day 1987, Chor and Tuller 2005, Fiorini and Joret 2012). Consequently, the most widely used solution approaches for these problems have relied on heuristic methods based on local search or Markov chain Monte Carlo (MCMC) sampling (Felsenstein 2003).

In these approaches, the tree space is explored iteratively by modifying the current tree through subtree rearrangement operations that alter the tree topology (Felsenstein 2003). This strategy underlies many widely used phylogenetic inference tools and has proven effective in practice, making such heuristics the backbone of modern phylogenetic analysis.

Nevertheless, the ability of these methods to reach optimal solutions or converge to the true posterior distribution remains uncertain, even for moderately sized datasets (Zhou *et al.* 2018, Fabreti and Höhna 2022, Liu *et al.* 2024, Spirin *et al.* 2024). This difficulty largely arises from the superexponential size and ruggedness of the solution space, together with a strong dependence on the initial solution.

Given the aforementioned challenges, alternative approaches to phylogenetic inference have been explored. The most prominent of them is Integer Linear Programming (ILP) (Gusfield 2019). ILP offers several advantages, including their ability to find optimal solutions, relative ease of expressing many problems in ILP form, and the availability of efficient commercial and open-source solvers. However, ILP-based methods usually face significant scalability limitations. This has motivated the development of LP relaxation-based approaches such as Catanzaro *et al.* (2025), in which integrality constraints are removed and the resulting fractional solutions are transformed into valid phylogenies by a rounding procedure.

In this study, we introduce an alternative mathematical programming approach to phylogenetic inference based on *Semidefinite Programming (SDP)*. SDP is a powerful optimization methodology that involves minimizing a linear objective function subject to linear constraints over the cone of positive semidefinite matrices (Vandenberghe and Boyd 1996). It is a nonlinear convex optimization problem that can be solved in polynomial time using interior-point methods. SDP generalizes LP and provides tight relaxations for a wide range of combinatorial optimization problems (Lovász 2003). It has been successfully applied in many fields, ranging from engineering (Wolkowicz *et al.* 2012) to quantum computing (Skrzypczyk and Cavalcanti 2023). Although SDP has been applied to haplotyping (Kalpakis and Namjoshi 2005), protein structure determination (Alipanahi *et al.* 2013), protein docking (Marcia *et al.* 2007) and phylogenetics (Snir and Rao 2010, Ben-Dor *et al.* 1998), its use in computational biology remains limited.

Here, we demonstrate that SDP relaxations can be used for phylogenetic inference. A phylogenetic tree induces a nested system of taxon partitions, suggesting a natural matrix-based representation of tree structure. This representation can be encoded using SDP variables and constraints, yielding a convex relaxation that can be efficiently rounded to a discrete tree solution.

As a proof of concept, we consider the phylogenetic inference problem under the Balanced Minimum Evolution (BME) model. The original problem formulation was based on the branch-length estimator of Pauplin (2000) and seeks a phylogeny minimizing a weighted least-squares discrepancy between a dissimilarity matrix *D* and tree distances, with weights decreasing exponentially in the number of internal nodes separating taxa (Desper and Gascuel 2004). More recently, the BME objective was shown to admit an information-theoretic interpretation as the minimization of a cross-entropy between path-length distributions induced by the phylogeny and probability distributions derived from the observed dissimilarity matrix (Catanzaro *et al.* 2020a).

The BME model is one of the most widely used approaches in distance-based phylogenetics. It has been extensively studied theoretically (Pauplin 2000, Desper and Gascuel 2002, 2004, Semple and Steel 2004, Forcey *et al.* 2016), implemented in widely used software tools (Lefort *et al.* 2015), and applied in numerous empirical studies (Edger *et al.* 2019, Librado *et al.* 2021, Wick *et al.* 2021). The model also has several important features: it is statistically consistent (Desper and Gascuel 2004, Semple and Steel 2004); the classical Neighbor-Joining (NJ) algorithm is essentially as a greedy heuristic for optimizing the BME objective (Gascuel and Steel 2006); and in practice it often achieves higher accuracy than alternative phylogenetic methods on real data (Spirin *et al.* 2024). For a comprehensive review of the BME problem, see Catanzaro *et al.* (2022).

BME tree inference problem is NP-hard and, moreover, cannot be approximated within a factor of $c^{n}$ for a constant c>1 (Fiorini and Joret 2012), even when the topology of the binary tree is fixed and only the taxa assignment to leaves remains to be determined (Frohn 2021). Consequently, a variety of exact and heuristic algorithmic approaches have been explored, including local search (Desper and Gascuel 2002, Lefort *et al.* 2015, Gasparin *et al.* 2023) and mathematical programming (Catanzaro *et al.* 2012, 2020b, 2025, 2026, Forcey *et al.* 2016).

This work continues research within the latter category, alongside recent advances in LP-relaxation methodology (Catanzaro *et al.* 2025). We propose a novel algorithmic approach to BME problem based on solving an SDP relaxation followed by rounding scheme for transforming relaxed solutions to actual tree solutions. The relaxation preserves the dyadic weighting structure of BME, captures hierarchical nesting of clades through semidefinite constraints, and remains convex, enabling efficient solution via interior-point methods. Using simulated and empirical data, we demonstrate that the resulting algorithm, which we call *SDPTree*, allows for accurate phylogenetic reconstruction on moderately sized inputs. The proposed scheme is general enough and potentially extendable to other phylogenetic inference problems.

## 2 Methods

### 2.1 Matrix formulation of the BME problem

Consider n≥3 taxa together with their n×n dissimilarity matrix $D={\left(d_{ij}\right)}_{i,j=1}^{n}$. Let [n]={1,…,n}, and let $T_{n}$ denote the set of all full binary phylogenetic trees (i.e. trees in which every vertex has degree either 1 or 3) with leaf set *[n]*. The Balanced Minimum Evolution (BME) problem can be formulated as


(1) $$\underset{T\in T_{n}}{\min}F(T)=\sum_{1\leq i,j\leq n}d_{ij}2^{-\tau_{ij}},$$

where $\tau_{ij}$ denotes the topological distance between leaves *i* and *j*, i.e. the number of edges on the unique (i,j)-path in a tree *T*.

For our purposes, we derive an equivalent formulation in which the objective is written in matrix form. The formulation is most naturally defined for a rooted tree; the unrooted objective can be obtained from the rooted one by a simple adjustment (see below).

Let *T* be a rooted tree with a vertex set V(T) and edge set E(T). We denote by $\rho_{u}$ the depth (distance from the root) of a vertex u∈V(T). Let $\sigma_{ij}$ be the depth of the lowest common ancestor of leaves i,j∈[n], and let $K={\text{max}}_{i\in [n]}\rho_{i}$ denotes the height of the tree. Then


(2) $$\tau_{ij}=\rho_{i}+\rho_{j}-2\sigma_{ij}$$

and the formulation (1) becomes


(3) $$\underset{T\in T_{n}}{\min}F(T)=\sum_{1\leq i,j\leq n}d_{ij}2^{2\sigma_{ij}}2^{-\rho_{i}}2^{-\rho_{j}}.$$

Using the notation $\zeta =\zeta_{T}={\left(2^{-\rho_{i}}\right)}_{i=1}^{n}$ and $\Sigma =\Sigma_{T}={\left(4^{\sigma_{ij}}\right)}_{i,j=1}^{n}$, the formulation (3) can be written as


(4) $$\underset{T\in T_{n}}{\min}F(T)=\langle D,\zeta_{T}\zeta_{T}^{⊤}{}^{\circ}\Sigma_{T}\rangle .$$

Here 〈A,B〉 denotes the matrix inner product and ◦ the Hadamard (element-wise) product. The variables $\zeta_{i}$ will be referred to as *dyadic weights*. In what follows, we omit the subscript *T* whenever the underlying tree is clear from the context.

Denote by $S^{n}$ the set of real symmetric n×n matrices. A matrix $M\in S^{n}$ is Positive SemiDefinite (PSD, denoted M≽0) whenever any of the following equivalent conditions holds: (a) $w^{⊤}\mathrm{Mw}\geq 0$ for all $w\in R^{n}$; (b) M is a *Gram matrix*, i.e. $M=L^{⊤}L$ for some $L\in R^{m\times n}$; (c) all eigenvalues of M are nonnegative.

Let $R_{k}=\{u\in V(T):\rho_{u}=k\}$ be the set of vertices at depth *k*; each vertex $u\in R_{k}$ defines a cluster $L_{u}$ of its descendant leaves. Consider the sequence of matrices $B^{\left(0\right)},\ldots ,B^{\left(K\right)}$, where $B_{ij}^{\left(k\right)}$ is the indicator of whether *i* and *j* belong to the same cluster at level *k*:


(5) $$B_{ij}^{\left(k\right)}=\{\begin{array}{cc} 1, & \;\text{if}\;\sigma_{ij}\geq k,\;\;i,j\in [n],k\in [K]\\ 0, & \text{otherwise}. \end{array}$$

In particular, $B^{\left(0\right)}=1_{n}1_{n}^{⊤}$ is the all-ones matrix.

Lemma 1 .

*The following relations hold:*

*(i)* $B^{\left(k\right)}≽0$  *for* k=0,…,K.

*(ii)* $\Sigma =\sum_{k=0}^{K}\beta_{k}B^{\left(k\right)}≽0$*, where* $\beta_{0}=1$  *and* $\beta_{k}=3\cdot 4^{k-1}$  *for* k∈[K].

Proof. For each *k*, let $U^{\left(k\right)}$ be the $|R_{k}|\times n$ matrix whose rows are the indicator vectors of clusters $L_{u}$, $u\in R_{k}$. Then $B^{\left(k\right)}={\left(U^{\left(k\right)}\right)}^{⊤}U^{\left(k\right)}$, and hence $B^{\left(k\right)}≽0$.

To prove (ii), recall that by the definition the entries $B_{ij}^{\left(k\right)}$ are equal to one exactly for levels $k\leq \sigma_{ij}$ and are zero for $k>\sigma_{ij}$. Therefore


(6) $$\Sigma_{ij}=4^{\sigma_{ij}}=1+\sum_{k=1}^{\sigma_{ij}}\left(4^{k}-4^{k-1}\right)=1+\sum_{k=1}^{K}\left(4^{k}-4^{k-1}\right)B_{ij}^{\left(k\right)}$$

In a matrix form, this gives a telescopic representation


(7) $$\Sigma =B^{\left(0\right)}+\sum_{k=1}^{K}\left(4^{k}-4^{k-1}\right)B^{\left(k\right)}=\sum_{k=0}^{K}\beta_{k}B^{\left(k\right)}.$$

Thus Σ is a positive linear combination of PSD matrices and hence PSD. ▪

Let ZB denote the set of pairs (ζ,B), where $\zeta \in R^{n}$ is a dyadic vector and $B=(B^{\left(0\right)},\ldots ,B^{\left(K\right)})$ is a sequence of n×n symmetric binary matrices, that encode full binary trees. Necessary and sufficient conditions characterizing ZB are given in Supplemental Lemma S1. Using Lemma 1, the rooted BME problem can be formulated as


(8) $$\underset{(\zeta ,B)\in ZB}{\min}F(T)=\sum_{k=0}^{H}\beta_{k}\langle D,\zeta \zeta^{⊤}◦B^{\left(k\right)}\rangle .$$

Since $B^{\left(k\right)}≽0$ and $\zeta \zeta^{⊤}≽0$, their Hadamard product is also PSD by the Schur product theorem; hence $\zeta \zeta^{⊤}◦B^{\left(k\right)}≽0$.

Suppose now that *T* is an unrooted binary tree, and $T^{\prime }$ is its rooting, i.e. a rooted tree obtained by subdividing an edge of *T* with a new root vertex and orienting all edges away from the root. The distances $\tau_{ij}$ and $\tau_{ij}^{\prime }$ in *T* and $T^{\prime }$ coincide unless *i* and *j* lie on different sides of the root, in which case $2^{-\tau_{ij}}=2\cdot 2^{-\tau_{ij}^{\prime }}$. Thus the unrooted objective is obtained by setting $\beta_{0}=\beta_{1}=2$ and using (8).

### 2.2 SDP relaxation of the BME problem

Recall that SDP is the problem of minimizing a linear function 〈C,X〉 of a PSD matrix variable X≽0 subject to linear constraints $\langle A_{i},X\rangle \leq b_{i}$, i∈[m]. Thus (8) is not a valid SDP objective, since both ζ and $B^{\left(k\right)}$ are variables. Therefore we introduce additional variables relaxing the products $\zeta \zeta^{⊤}◦B^{\left(k\right)}$.

#### 2.3 Variables

The SDP formulation uses three main families of variables:

-
$P\in {\left[0,1\right]}^{n\times K}$ : relaxed depth assignments. Each row represents a distribution over depths $\rho_{i}\in [K]$, i.e. $P_{i,d}=\text{Pr}(\rho_{i}=d)$.
-
$Z\in S^{n}$ : a matrix variable relaxing the rank-one matrix $\zeta \zeta^{⊤}$.
-
$Y^{\left(k\right)}\in S^{n}$ , k=0,…,K: level-wise PSD matrices approximating $\zeta \zeta^{⊤}◦B^{\left(k\right)}$ and encoding the hierarchical partition of taxa induced by the tree.

To simplify notation, we also introduce auxiliary variables expressible in terms of the main variables. These are not essential for the optimization itself but make the constraints more compact and easier to state.

-
$z\in R^{n}$ and $s\in R^{n}$: dyadic leaf weights and their second moments, approximating $z_{i}\approx \zeta_{i}=2^{-\rho_{i}}$ and $s_{i}\approx \zeta_{i}^{2}$. They are coupled with P via
  (9) $$z_{i}=E[2^{-\rho_{i}}]=\sum_{d=1}^{K}2^{-d}P_{i,d},$$
   
  (10) $$s_{i}=E[2^{-2\rho_{i}}]=\sum_{d=1}^{K}2^{-2d}P_{i,d}.$$
-
$z^{\left(k\right)},s^{\left(k\right)},q^{\left(k\right)}\in R^{n}$ : truncated moments restricted to tree levels ≥k:
  (11) $$z_{i}^{\left(k\right)}=E\left[2^{-\rho_{i}}I\{\rho_{i}\geq k\}\right]=\sum_{d=k}^{K}2^{-d}P_{i,d},$$
   
  (12) $$s_{i}^{\left(k\right)}=E\left[2^{-2\rho_{i}}I\{\rho_{i}\geq k\}\right]=\sum_{d=k}^{K}2^{-2d}P_{i,d},$$
   
  (13) $$q_{i}^{\left(k\right)}=E\left[I\{\rho_{i}\geq k\}\right]=\sum_{d=k}^{K}P_{i,d}.$$

Next, we describe the constraints.

**Probability distribution constraint:**


(14) $$P1_{K}=1_{n}$$

**Kraft constraints:**


(15) $$z^{⊤}1_{n}=1$$

(16) Z1=z,

(17) $$Y^{\left(k\right)}1_{n}=2^{-k}z^{\left(k\right)},\;k=0,\ldots ,K$$

Constraint (15) relaxes the Kraft equality $\sum_{i=1}^{n}\zeta_{i}=1$ for leaf depths in a full binary tree (Cover and Thomas 1999, Catanzaro *et al.* 2012). Constraint (16) relaxes its corollary $\sum_{j}\zeta_{i}\zeta_{j}=\zeta_{i}$ by replacing $\zeta_{i}\zeta_{j}$ and $\zeta_{i}$ with $Z_{ij}$ and $z_{i}$. Constraint (17) is the subtree version, $\sum_{j}B_{ij}^{\left(k\right)}\zeta_{j}=2^{-k}$, multiplied by $\zeta_{i}$ and relaxed via $B_{ij}^{\left(k\right)}\zeta_{i}\zeta_{j}\mapsto Y_{ij}^{\left(k\right)}$.

**PSD and non-negativity constraints**


(18) $$Y^{\left(k\right)},Z≽0,$$

(19) $$Y_{ij}^{\left(k\right)},Z_{ij}\geq 0\;i,j\in [n].$$

**Diagonal constraints**


(20) diag(Z)=s,

(21) $$\mathrm{diag}(Y^{\left(k\right)})=s^{\left(k\right)},\;k\in [K].$$

These relax the identities ${\left(\zeta \zeta^{⊤}\right)}_{ii}=2^{-2\rho_{i}}$ and ${\left(\zeta \zeta^{⊤}◦B^{\left(k\right)}\right)}_{ii}=2^{-2\rho_{i}}1\{\rho_{i}\geq k\}$.

**Cluster nestedness**


(22) $$Y_{ij}^{\left(k+1\right)}\leq Y_{ij}^{\left(k\right)}\;i,j\in [n],\;k=0,\ldots ,K-1.$$

This relaxes the requirement that clusters form a nested hierarchy: if two leaves are separated at level *k*, they remain separated at all deeper levels.

**Shor lifting**


(23) $$\begin{array}{cc} \left(\begin{array}{cc} 1 & z^{⊤}\\ z & Z \end{array}\right) & ≽\;0. \end{array}$$

(24) $$\begin{array}{cc} \left(\begin{array}{cc} q_{i}^{\left(k\right)} & z_{i}^{\left(k\right)}\\ z_{i}^{\left(k\right)} & s_{i}^{\left(k\right)} \end{array}\right) & ≽\;0,\;i\in [n],\;k=0,\ldots ,K. \end{array}$$

Constraint (23) is the *Shor semidefinite lifting* (Ben-Tal and Nemirovski 2001), equivalent via the Schur complement (Boyd *et al.* 2004) to $Z-zz^{⊤}≽\;0$, and thus a convex relaxation of $Z=zz^{⊤}$. Constraint (24) implies ${\left(z_{i}^{\left(k\right)}\right)}^{2}\leq q_{i}^{\left(k\right)}s_{i}^{\left(k\right)}$, the Cauchy–Schwarz inequality applied to (11)–(13).

**McCormick envelope**


(25) $$Z_{ij}\leq \frac{1}{2}z_{i}+\frac{1}{2^{K}}z_{j}-\frac{1}{2^{K+1}},$$

(26) $$Z_{ij}\leq \frac{1}{2^{K}}z_{i}+\frac{1}{2}z_{j}-\frac{1}{2^{K+1}},$$

(27) $$Z_{ij}\geq \frac{1}{2^{K}}z_{i}+\frac{1}{2^{K}}z_{j}-\frac{1}{2^{2K}},$$

(28) $$Z_{ij}\geq \frac{1}{2}z_{i}+\frac{1}{2}z_{j}-\frac{1}{4},1\leq i<j\leq n$$

Constraints (25)–(28) form the *McCormick envelope* (McCormick 1976), a standard convex relaxation for bilinear relations such as $Z_{ij}=z_{i}z_{j}$.

**Additional coupling constraints**


(29) $$Y^{\left(0\right)}=Z,$$

(30) $$Y_{ij}^{\left(k\right)}\leq Z_{ij},\;i,j\in [n],\;k=0,\ldots ,K,$$

(31) $$Z_{ij}\leq z_{i},\;Z_{ij}\leq z_{j},\;i,j\in [n].$$

Here, (29) relaxes $\zeta \zeta^{⊤}◦B^{\left(0\right)}=\zeta \zeta^{⊤}$ (since $B^{\left(0\right)}=1_{n}1_{n}^{⊤}$), while (30) reflects $Y_{ij}^{\left(k\right)}\approx \zeta_{i}\zeta_{j}B_{ij}^{\left(k\right)}\leq \zeta_{i}\zeta_{j}\approx Z_{ij}$.

In summary, the SDP relaxation of the BME problem is


(32) $$\text{min}\sum_{k=0}^{K}\beta_{k}\langle D,Y^{\left(k\right)}\rangle \;s.t.\;(9)—(31).$$

It is informative to compare (2) with the ILP formulations described in Catanzaro *et al.* (2012, 2020b, 2026). Both approaches encode tree structures and dyadic BME weights, but in different extended spaces: the ILP formulations use a direct integer encoding of path lengths, whereas (32) uses a semidefinite relaxation of a factorized depth/co-ancestry representation. In the integer case, the two encodings are connected through (2), and both involve Kraft-type consistency conditions. However, their constraints are based on different structural characterizations: the SDP formulation uses PSD, laminarity, and depth-consistency constraints for hierarchical cluster matrices, whereas the ILP formulations enforce path-length realizability through strong triangle inequalities and Buneman-type conditions (Buneman 1974).

This SDP formulation contains $O(n^{2}K)$ variables and constraints. For comparison, the basic LP formulation of Catanzaro *et al.* (2025) uses $O(n^{3})$ variables and constraints.

### 2.4 Rounding the relaxation

After solving the SDP relaxation, the fractional solution is converted into a binary tree. As noted above, the matrices $Y^{\left(k\right)}$ are convex relaxations of the exact relations $Y^{\left(k\right)}=Z◦B^{\left(k\right)}$. While the SDP constraints enforce PSDness, nestedness, Kraft-type depth constraints, and consistency with the leaf-depth variables, they do not fully characterize tree-realizable cluster matrices $B^{\left(k\right)}$. Consequently, the feasible region of SDP relaxation extends beyond the convex hull of tree-realizable solutions, and fractional solutions may distribute co-membership mass among multiple clusters. This makes one-shot rounding unstable, motivating an agglomerative scheme that resolves the hierarchy gradually by committing only to the most strongly supported local merges.

Specifically, at each rounding iteration we identify, from the SDP solution, the *L* pairs of taxa most likely to form cherries, merge them into their parents, update the objective accordingly, and repeat the SDP + cherries detection procedure for n−L taxa until the full tree *T* is obtained. A similar approach for an ILP relaxation was independently proposed in Catanzaro *et al.* (2025).

To estimate likely cherries we use two approaches, further referred to as *separability* and *profile* rounding heuristics, respectively. In the separability approach, we recover relaxed clade co-membership matrices $B^{\left(k\right)}$ via $B_{ij}^{\left(k\right)}=Y_{ij}^{\left(k\right)}/\sqrt{Z_{ii}Z_{jj}}$. We say that taxa *i* and *j* are *strongly inseparable* at the level *k*, if $B_{ij}^{\left(k\right)}={\text{max}}_{u\neq v}B_{uv}^{\left(k\right)}\}$. Then we consider the weighted graph $G_{C}$, with weights $C_{ij}$ of edges *ij* being the number of levels at which *i* and *j* are strongly inseparable. We find the maximum-weight matching $\left(i_{1},j_{1}),\ldots ,(i_{L^{\prime }},j_{L^{\prime }}\right)$ of the size at most *L* in $G_{C}$, and merge the pairs $\left(i_{l},j_{l}\right)$, $l\in [L^{\prime }]$.

Alternatively, we consider the matrix $\Delta =\sum_{k=0}^{K}\beta_{k}Y^{\left(k\right)}$. By (7), for binary solutions


(33) $$\Delta_{ij}=\sum_{k=0}^{K}\beta_{k}\zeta_{i}\zeta_{j}B_{ij}^{\left(k\right)}=\zeta_{i}\zeta_{j}\Sigma_{ij}=2^{-\tau_{ij}},$$

so $\Delta_{ij}$ relaxes $2^{-\tau_{ij}}$. We therefore consider the weighted graph $G_{\Delta }$ with edges weights $w_{ij}=\|\Delta_{i,:}-\Delta_{j,:}{\|}_{1}$ measuring the similarity between distance profiles of each leaf pair to the remaining leaves. Then we merge the pairs $\left(i_{l},j_{l}\right)$, l∈[L] with most similar distance profiles, i.e. those forming the minimum-weight *L*-matching in $G_{\Delta }$.

The selected pair (i,j) is replaced by its parent $p_{ij}$, and the dissimilarity matrix D is updated according to the BME objective by replacing the *i*th and *j*th rows and columns with their average.

The matching size *L* is the algorithm parameter with the default value L=1; In our implementation minimum or maximum *L*-matchings are estimated using the simple greedy algorithm. In practice, we apply both separability and profile rounding heuristics and select the better of the two resulting trees, which is then refined using Subtree Pruning and Regrafting (SPR) local search.

The full BME inference algorithm called SDPTree was implemented in Matlab R2024b (MathWorks Inc.). The SDP relaxation was solved using MOSEK 11.1 (Mosek ApS) via the YALMIP toolbox (Löfberg 2004). Calculations were performed on a workstation with Intel Xeon Platinum 8268 processor (2.90 GHz, 96 cores) and 256 GB of RAM under Ubuntu 22.04.5 operating system.

## 3 Results

### 3.1 Data and algorithms

The proposed algorithm was validated and benchmarked on simulated and real data. In all experiments, the input consisted of randomly generated dissimilarity matrices $D\in R^{n\times n}$ with n=10,15,…,50, produced by different models. We used four types of simulated data:

1. Hamming distance matrices between *n* binary sequences of length *m*. In each experiment, a phylogenetic tree topology was generated using the coalescent model (Kingman 1982), and sequences were simulated along its branches with substitution rate *p* under the Camin–Sokal model (Camin and Sokal 1965).
2. Euclidean distance matrices for *n* points randomly sampled from $R^{m}$.
3. Random Doubly Stochastic Matrices (RDSM) with zero diagonals, publicly available from Catanzaro *et al*. (2025).
4. Random Integer Matrices (RIM), also used in Catanzaro *et al*. (2025).

In both (1) and (2) we used m=1000, roughly matching the order of magnitude typical for targeted amplicon or single-cell sequencing (Kozlov *et al.* 2022, Lin *et al.* 2024).

For empirical data validation we used the PhyloBench dataset (Spirin *et al.* 2024), that was specifically designed for phylogenetic benchmarking. It contains 5847 benchmark instances, each with n=15,30, or 45 sequences from orthologous protein domains randomly sampled from several thousand orthologous groups across 12 taxonomic groups spanning Bacteria, Archaea, and Eukaryota.

In all computational experiments, SDP relaxations were solved using MOSEK with default conic interior-point tolerances of $10^{-8}$ and infeasibility detection tolerance of $10^{-12}$.

The following algorithms were selected for comparison:

1. The Neighbor-Joining (NJ) algorithm (Saitou and Nei 1987), which, as noted above, is an agglomerative heuristic for the BME problem (Gascuel and Steel 2006).
2. Methods employing SPR-based local search. We used two variants of the algorithm, initialized with either a random phylogeny or an NJ-reconstructed phylogeny.
3. The LP-relaxation-based method BeamLPNJ (Catanzaro *et al*. 2025). For a fair comparison, we used settings analogous to those employed for SDPTree: (i) beam width B=1; and (ii) the better of the two algorithm variants, with and without triangle inequalities in the LP formulation, analogous to selecting the better of the separability and profile rounding schemes in SDPTree.

### 3.2 Computational experiments

In the first series of experiments, we compared different parameterizations of the SDP relaxation and rounding procedures (prior to SPR adjustment). We considered both linear (K=n/2) and logarithmic (K=2 log n) upper bounds on the tree height in the SDP formulation. In the logarithmic setting, we further evaluated different matching sizes (L=1,2,4,6,8) used during the merging stage.

**Figure 1:**
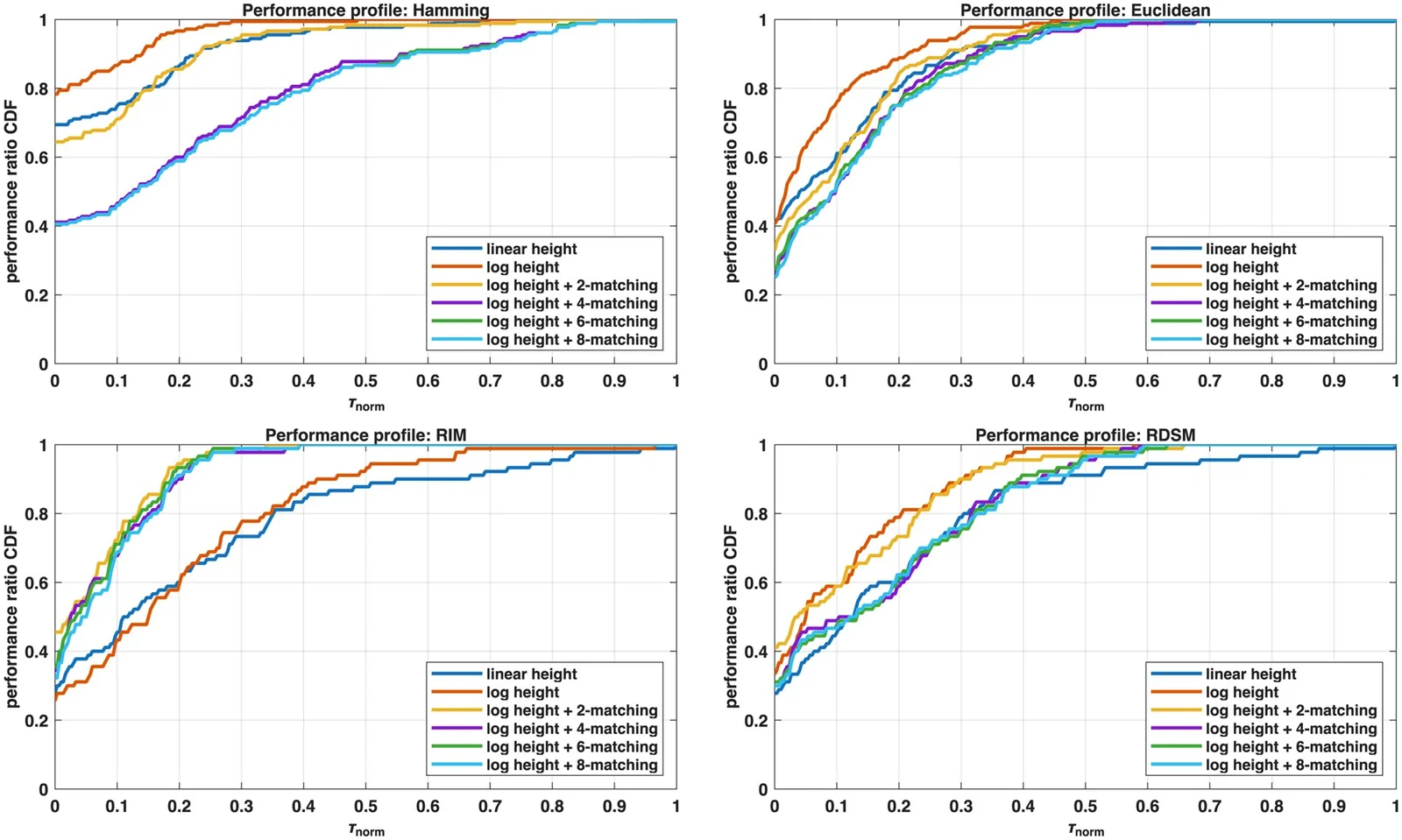
Performance profiles of SDP relaxation and rounding under different algorithm settings. X-axis represents normalized performance ratio values $\tau_{\mathrm{norm}}=\frac{\tau -1}{\tau_{\text{max}}-1}$, $1\leq \tau \leq \tau_{\text{max}}$.

To compare algorithm performance, we used Dolan–Moré performance profiles (Dolan and Moré 2002) (Fig. 1), defined as follows. Given a set of solvers S and test instances I, the performance ratio of solver S∈S on instance I∈I is


$$r_{SI}=\frac{F_{SI}}{{\text{min}}_{S^{\prime }\in S}F_{S^{\prime }I}},$$

where $F_{SI}$ is the objective value returned by *S*. The performance profile


$$\Phi_{S}(\tau )=\frac{1}{\left|I\right|}\sum_{I\in I}1\{r_{SI}\leq \tau \}$$

is the cumulative distribution function of these ratios over I. To compare profiles quantitatively, we use the area under the curve (AUC)


(34) $${\text{AUC}}_{S}=\frac{1}{\tau_{\text{max}}-1}\int_{1}^{\tau_{\text{max}}}\Phi_{S}(\tau )d\tau ,$$

where $\tau_{\text{max}}$ is the maximum ratio across all solvers (Table 1).

**Table 1:** AUC for performance profiles of SDP relaxation and rounding under different algorithm settings.

	Hamming	Euclidean	RIM	RDSM
Linear height	0.933	0.895	0.793	0.816
Log height	0.972	0.934	0.814	0.895
Log height + 2-matching	0.928	0.898	0.938	0.887
Log height + 4-matching	0.799	0.875	0.929	0.838
Log height + 6-matching	0.792	0.877	0.932	0.836
Log height + 8-matching	0.791	0.870	0.925	0.835

We observed that a conservative linear upper bound on tree depth, in addition to being more computationally intensive, generally leads to lower accuracy than a logarithmic bound, with the latter producing higher performance profiles AUC for all four simulated data types (Fig. 1 and Table 1). This can be explained by the fact that bounding the depth *K* in the SDP relaxation acts as a form of regularization: smaller *K* limits how small the dyadic weights $\zeta_{i}$ can become, reducing the model’s dynamic range and improving conditioning. It also restricts the relaxation’s ability to hide inconsistencies by pushing leaves arbitrarily deep, while fewer levels limit the accumulation of slack and relaxation looseness from nestedness and balance constraints, yielding solutions that are more coherent and easier to round. It also should be noted that limiting *K* improves relaxation and rounding, while still allowing recovery of deeper or less balanced trees during agglomeration when warranted by the data.

The effect of the matching size *L* (the number of leaves merged at each agglomeration step) is less predictable and more data-dependent, as merging multiple cherries can have both positive and negative effects (Fig. 1 and Table 1). When the SDP produces well-separated candidates, merging several cherries in one step is beneficial: it performs multiple contractions jointly and is largely order-insensitive, avoiding a common failure mode of sequential greedy merging, where an early suboptimal choice prevents a later correct merge. Conversely, when cherry scores are ambiguous (i.e. have small margins or competing partners), merging many at once can reduce accuracy by committing to several uncertain contractions before re-solving the SDP. This limits the effect of the updated input and can amplify the impact of incorrect merges.

The above relationships between algorithm settings are largely consistent across input sizes (Figs. S1–S3).

**Figure 2:**
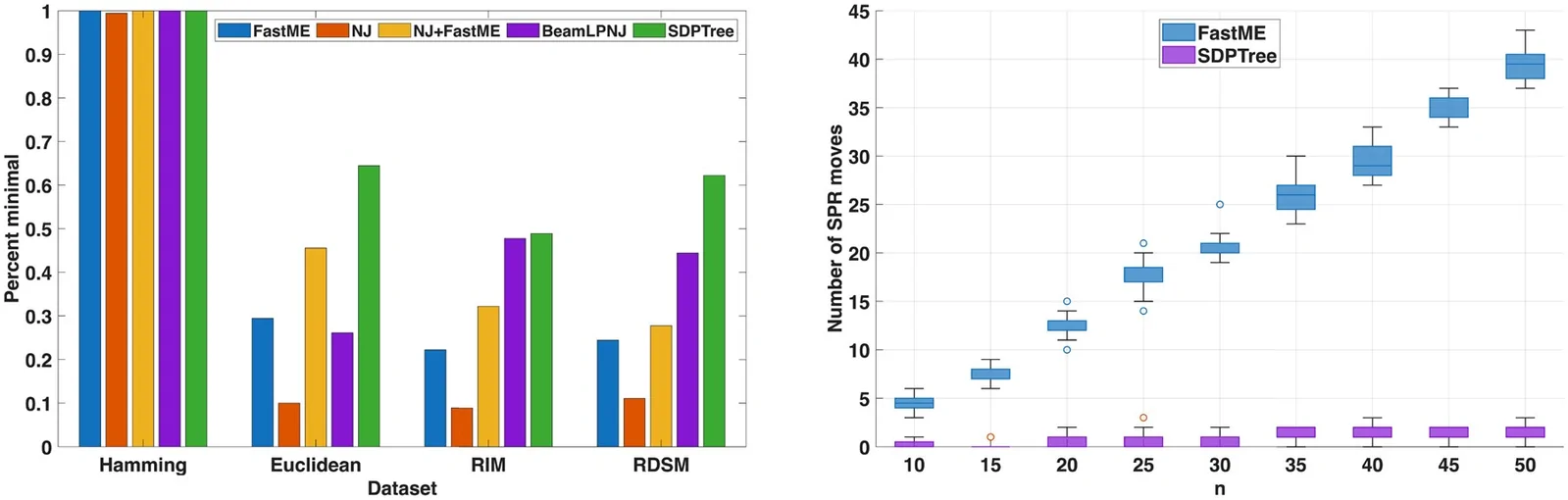
Left: Joint comparison of four methods on simulated data. The y-axis shows the percentage of instances where a method achieved the best objective value among the four methods (ties allowed). Right: Number of SPR moves for FastME (random initial tree) and SDPTree after SDP rounding for the Hamming dataset.

**Figure 3:**
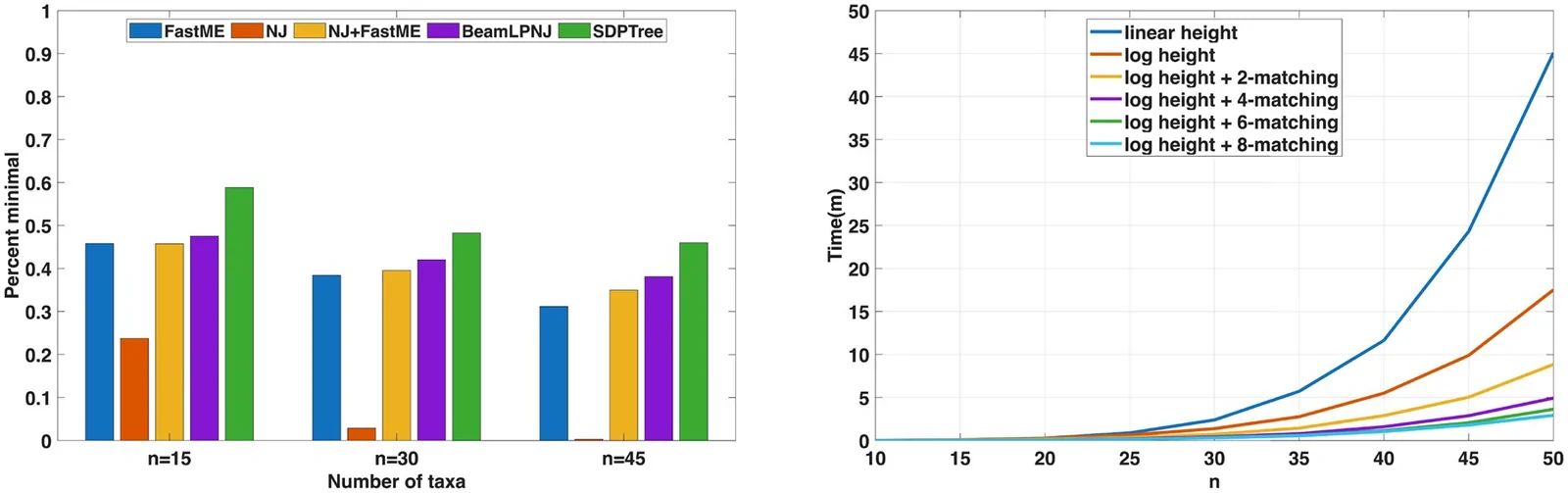
Left: Joint comparison of four methods on Phylobench data. The y-axis shows the percentage of instances where a method achieved the best objective value among the four methods (ties allowed). Right: running time of SDPTree (in minutes).

In the next series of experiments, we compared SDPTree with other methods. Based on the above results and the trade-off between solution quality and running time (Fig. 3, right), we selected the logarithmic depth bound with L=2 for benchmarking. Overall, SDPTree consistently outperformed competing methods across simulation settings (Fig. 2, left). For instance, in pairwise comparison with the most widely used method (FastME with an NJ initial tree), SDPTree produced better, identical, and worse solutions in 58.6%, 22.5%, and 18.9% of cases, respectively (*P* < 10^−3^, two-sided sign test, Table S1). The advantage of SDPTree tends to increase with input size (Fig. S4), with the above percentages going from (10%,87.5%,2.5%) for n=10 to (80%,0%,20%) for n=50.

The exception is the Hamming distance dataset (Fig. 2, left), where all four algorithms almost always returned identical objective values; this dataset was specifically designed to verify such behavior. Here, the substitution rate was set to *P* = .05, yielding nearly additive matrices *D* (with quartet splits matching the generating tree in 99.2% of cases on average). It is known that in such settings NJ with and without SDP adjustment produces optimal or near-optimal solutions (Atteson 1999). The experiments confirm that SDP relaxation with rounding preserves this guarantee. Indeed, SDP rounding landed very close to the optimal solution without SPR in almost all cases (Fig. 2 right): refinement required at most 2 SPR moves in 96.8% of cases, including 0 moves in 39.4% and 1 move in 42.2%. The number of moves was nearly independent of input size and significantly lower than for SPR with a random initial tree (*P* < 10^−40^, two-sided sign test; Fig. 2 right).

A similar pattern was observed for Phylobench data (Fig. 3, left, Tables 2 and S2). Across all three problem sizes, the SDPTree consistently outperforms the benchmark baselines, achieving the best objective value on the largest share of test instances and maintaining this advantage as *n* increases (*P* < 10^−84^, two-sided sign test for SDPTree versus other algorithms). Overall, these results indicate that the SDP relaxation captures informative global structure that translates into high-quality trees after rounding, and, from the optimization perspective, the resulting solutions are superior to standard heuristics and local search baselines across the tested sizes.

**Table 2:** AUC for performance profiles of SDP relaxation and rounding under different algorithm settings.

	Hamming	Euclidean	RIM	RDSM	Phylobench
FastME	0.998	0.696	0.683	0.715	0.811
NJ	0.993	0.475	0.423	0.484	0.252
NJ + FastME	0.998	0.836	0.772	0.768	0.837
BeamLPNJ	0.998	0.792	0.898	0.909	0.889
SDPTree	0.998	0.940	0.904	0.933	0.912

## 4 Discussion

In this study, we proposed a novel algorithmic approach to phylogenetic inference based on Semidefinite Programming (SDP) and applied it to the Balanced Minimum Evolution (BME) problem, one of the most widely used distance-based models. To the best of our knowledge, this is among the first attempts to harness SDP, that has previously been successful in combinatorial optimization, for computational phylogenetics.

Our benchmarking results demonstrate the higher accuracy of the SDP-based approach in comparison with several existing methods. These results should, however, be interpreted in the context of the known algorithmic properties of BME. The exponential inapproximability of the problem (Fiorini and Joret 2012, Frohn 2021) limits the prospects for obtaining provable approximation guarantees from SDP relaxations. Rather than approximating the objective within a worst-case factor, the relaxation serves as a heuristic mechanism for extracting structural information that guides the search toward high-quality regions of tree space. Empirically, the rounded SDP solutions are often already close to a local optimum, requiring only a few SPR moves to reach a locally optimal tree.

The semidefinite relaxation strategy based on cut-based constraints developed here can potentially be transferred to other phylogenetic optimization problems. Many objectives of interest in phylogenetics can be expressed (exactly or approximately) as linear or convex functions of pairwise quantities induced by a tree, while the main combinatorial difficulty lies in enforcing that the underlying representation is consistent with a valid tree topology. In such settings, one can introduce lifted matrix variables encoding co-membership or split structure, impose tractable convex constraints capturing necessary properties of trees (e.g. hierarchical nesting/laminarity, balance or capacity conditions, and metric or ultrametric inequalities), and then tighten the relaxation by adding valid linear or conic cuts. This paradigm suggests SDP-based bounding and rounding frameworks for related minimum-evolution variants, least-squares tree fitting, quartet-consistency formulations, and ultrametric (molecular clock) models, where the relaxation provides both certificates of optimality gaps and informative fractional structure that can be exploited by downstream rounding procedures.

There is, however, room for improvement. The main limitation of the SDP approach, as with many mathematical programming methods, is running time and memory consumption: the relaxation introduces multiple positive semidefinite matrix variables, making generic interior-point methods expensive. Moreover, the relaxation may remain loose in regimes where the data are noisy or the depth/topology variables provide many degrees of freedom, leading to fractional solutions whose hierarchical signal is diffuse and therefore harder to round reliably. As a result, the current SDP relaxation is not yet suitable for large inputs. This appears to be a common limitation of mathematical programming approaches to BME, including both SDP- and LP-based formulations (Catanzaro *et al.* 2025).

Several directions could improve both tightness and scalability:

- developing stronger, problem-specific cuts (e.g. additional laminarity/compatibility constraints, objective-aware inequalities, and tighter envelope constraints linking matrix variables), so that tighter bounds can be obtained without uniformly inflating the model size;
- using warm starts and model reuse to accelerate repeated solves in agglomerative steps, including adaptive stop tolerances tuned to rounding quality;
- replacing the agglomerative leaf-merging scheme by a single SDP solve followed by SDP-based randomized projection rounding (e.g. Goemans–Williamson–type hyperplane rounding) producing a sequence of bipartitions that can be assembled into an unrooted tree, possibly repeated to sample multiple candidate solutions.
- considering multiple alternative merges at each agglomerative iteration, similarly to beam search used in Catanzaro *et al*. (2025).

Implementing these improvements, as well as extending the SDP-based framework to related phylogenetic inference objectives, are promising directions for future research.

Another important avenue for future research is theoretical analysis of the SDP relaxation, including establishment of integrality gaps and corresponding tightness results (Atteson 1999), as well as comparison with LP relaxations derived from known BMEP polytope facets (Catanzaro *et al.* 2026).

## Supplementary Material

btag591_Supplementary_Data
