# Supplementary material for: Phylogenetic inference under the balanced minimum evolution criterion via semidefinite programming
Source: Bioinformatics. 2026 Aug 6;42(8):btag591. doi: 10.1093/bioinformatics/btag591 (PMC13476081; doi:10.1093/bioinformatics/btag591)
Supplement: Supplementary file 1 [file btag591_supplementary_data.pdf]

Supplement

P. Skums[1,†]

[1]School of Computing, University of Connecticut, Storrs, CT, USA
[†]Email: pavel.skums@uconn.edu

Lemma S1. *Let $\zeta \in \mathbb{R}^n_{\geq 0}$ be a dyadic vector, and $B^{(0)}, ..., B^{(n-1)}$ be a sequence of symmetric binary matrices. Then $\zeta, B^{(0)}, ..., B^{(n-1)}$ encode a full binary tree if and only if the following conditions hold:*

1) $B^{(0)} = \mathbf{1}_n \mathbf{1}_n^\top$.
2) $\sum_{j: B^{(k)}_{ij}=1} \zeta_j = 2^{-k}$, *for any* $k = 0, ..., n-1$ *and* i *such that* $\zeta_i \leq 2^{-k}$.
3) $B^{(k)}_{ii} = \mathbb{I}\{\zeta_i \leq 2^{-k}\}$ *for any* $k = 0, ..., n-1$ *and* $i = 1, ..., n$.
4) $B^{(k)}_{ij} \leq B^{(k)}_{ii}$, $B^{(k)}_{ij} \leq B^{(k)}_{jj}$ *for any* $k = 0, ..., n-1$ *and* $i, j = 1, ..., n$.
5) $B^{(k)}_{ij} + B^{(k)}_{jl} - B^{(k)}_{il} \leq 1$, *for any* $k = 0, ..., n-1$ *and* $i, j, l = 1, ..., n$.
6) $B^{(k+1)}_{ij} \leq B^{(k)}_{ij}$ *for all* $k = 0, ..., n-2$ *and* $i, j = 1, ..., n$.

Proof. Suppose first that ζ and $B^{(k)}$, $k = 0, \ldots, n-1$, arise from a full binary tree T. Conditions 1), 3), and 4) follow directly from the definition of the matrices $B^{(k)}$. Condition 2) follows from the Kraft equality for the leaf depths of T [1, 2], applied to each subtree of T. Condition 5) expresses the transitivity of the relation "to belong to the same subtree rooted at level k." Finally, condition 6) states that if two leaves belong to the same subtree at level $k+1$, then they also belong to the same subtree at level k, which is exactly the nesting property of subtrees across consecutive levels. Hence, conditions 1)–6) are necessary.

To prove sufficiency, define binary relations $\sim_0, \ldots, \sim_{n-1}$ on the set of taxa by $i \sim_k j$ whenever $B^{(k)}_{ij} = 1$. Conditions 3)–5) imply that, for each k, these relations are equivalence relations.

Let $C^{(k,1)}, \ldots, C^{(k,L_k)}$ be the equivalence classes of $\sim_k$. Construct a directed graph T whose vertices are these equivalence classes, with an arc from $C^{(k,u)}$ to $C^{(k+1,v)}$ whenever $C^{(k,u)} \supseteq C^{(k+1,v)}$. By construction, T is a directed acyclic graph. Moreover, condition 6) implies that every equivalence class at level $k+1$ is contained in an equivalence class at level k. Since equivalence classes at the same level are pairwise disjoint, every vertex $C^{(k+1,v)}$ has a unique parent. Together with condition 1), which gives a unique root at level 0, this implies that T is a rooted tree. The singleton equivalence classes are the leaves of T. By condition 3), the singleton class $\{i\}$ has depth d_i satisfying $\zeta_i = 2^{-d_i}$.

To show that T is the desired tree, it remains to prove that it is binary. Consider a vertex $C^{(k,u)}$ and let $C^{(k+1,v_1)}, \ldots, C^{(k+1,v_r)}$ be its children. By condition 2),

$$\sum_{i \in C^{(k,u)}} \zeta_i = 2^{-k}, \qquad \sum_{i \in C^{(k+1,v_s)}} \zeta_i = 2^{-(k+1)}, \quad s = 1, \ldots, r. \tag{1}$$

Since the children form a disjoint partition of $C^{(k,u)}$, that is, $C^{(k,u)} = C^{(k+1,v_1)} \sqcup \cdots \sqcup C^{(k+1,v_r)}$, it follows that

$$2^{-k} = \sum_{i \in C^{(k,u)}} \zeta_i = \sum_{s=1}^{r} \sum_{i \in C^{(k+1,v_s)}} \zeta_i = r 2^{-(k+1)}. \tag{2}$$

Hence $r = 2$. Therefore every internal vertex of T has exactly two children, and thus T is a full binary tree. This completes the proof.

□

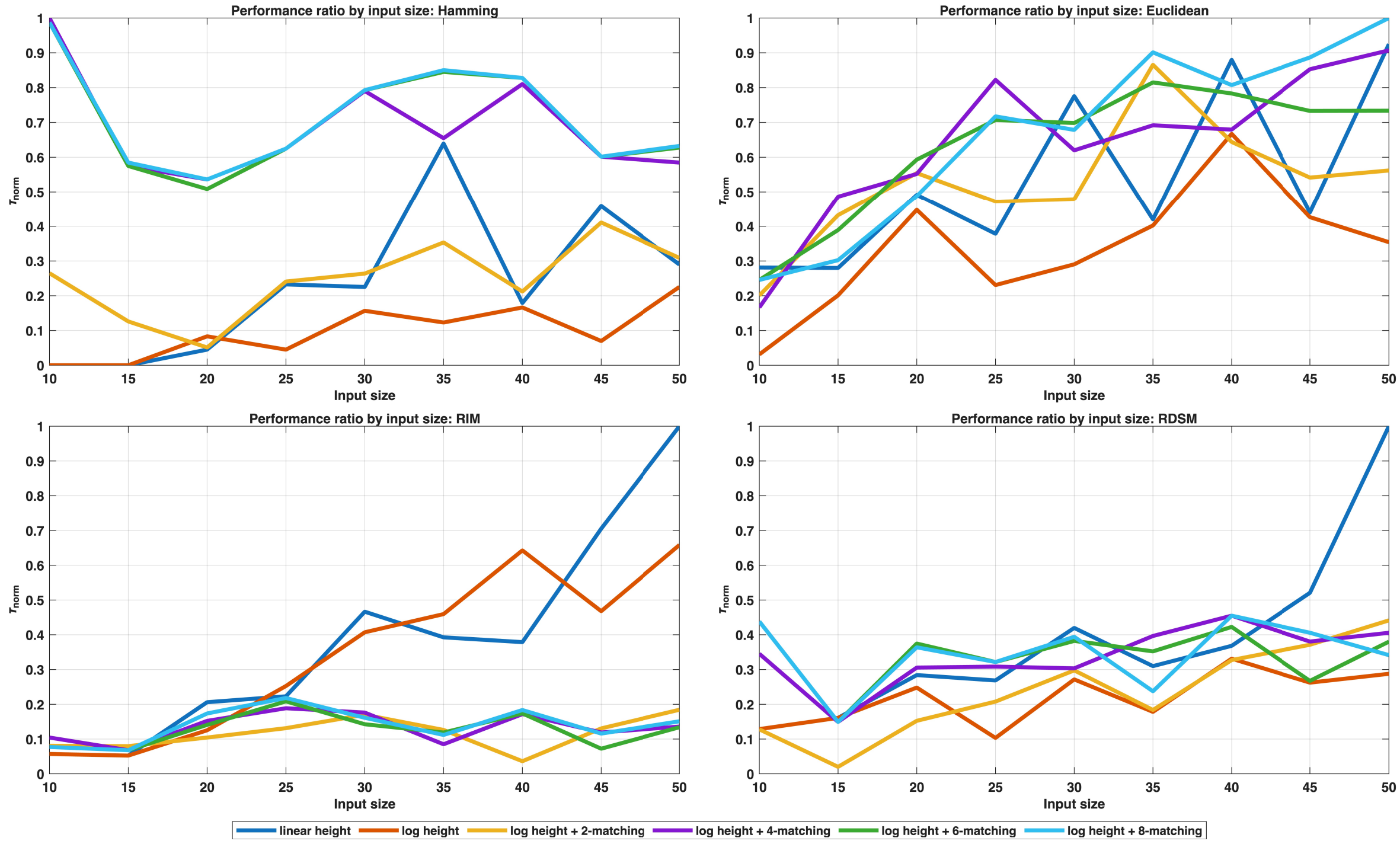

Figure S1: Mean performance ratios of SDP relaxation and rounding under different algorithm settings. X-axis represents input sizes, and Y-axis represents normalized performance ratio values $\tau_{norm} = \frac{\tau-1}{\tau_{\max}-1}$, $1 \leq \tau \leq \tau_{\max}$.

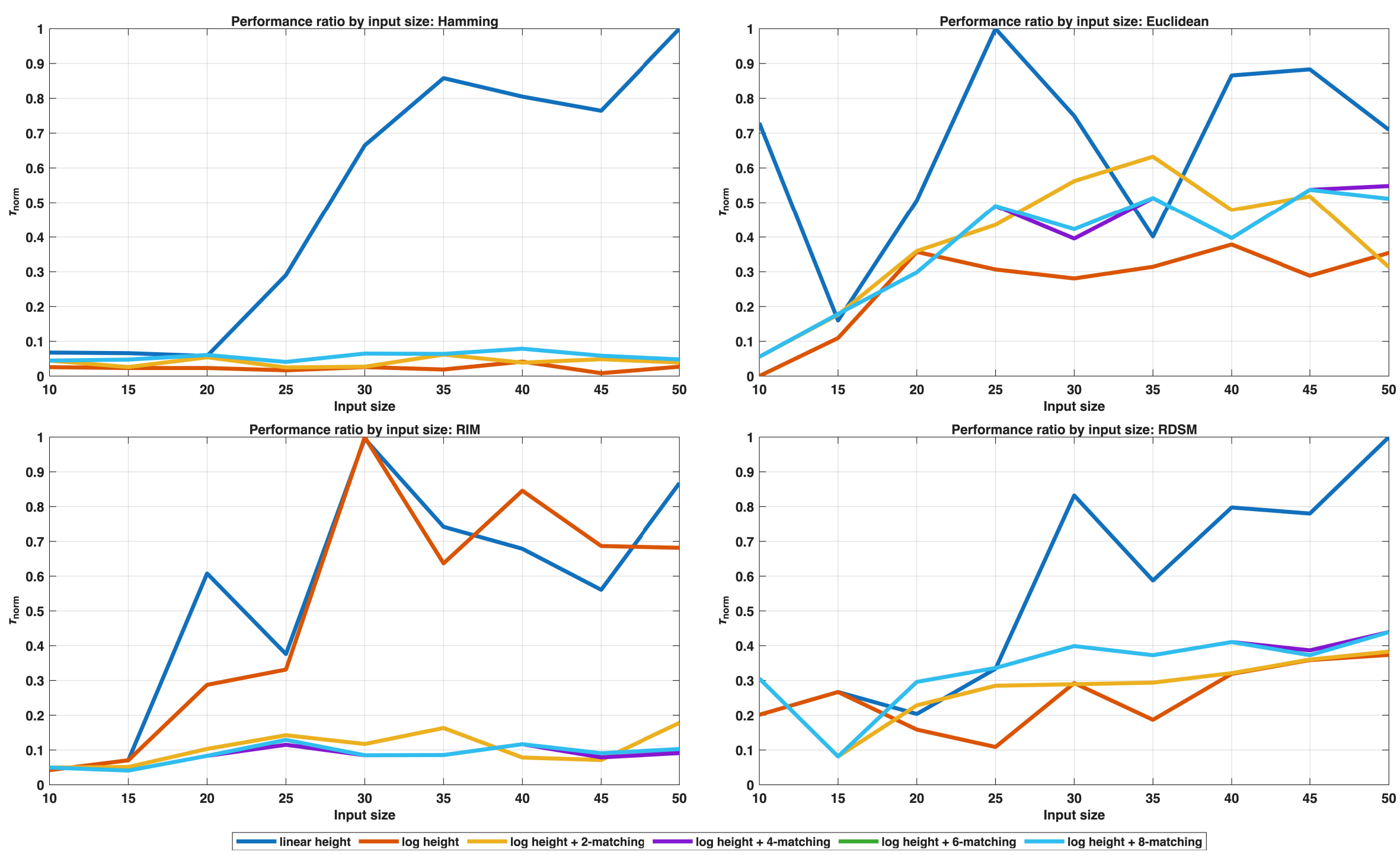

Figure S2: Mean performance ratios of SDP relaxation and separability rounding under different algorithm settings. X-axis represents input sizes, and Y-axis represents normalized performance ratio values $\tau_{norm} = \frac{\tau-1}{\tau_{\max}-1}$, $1 \leq \tau \leq \tau_{\max}$.

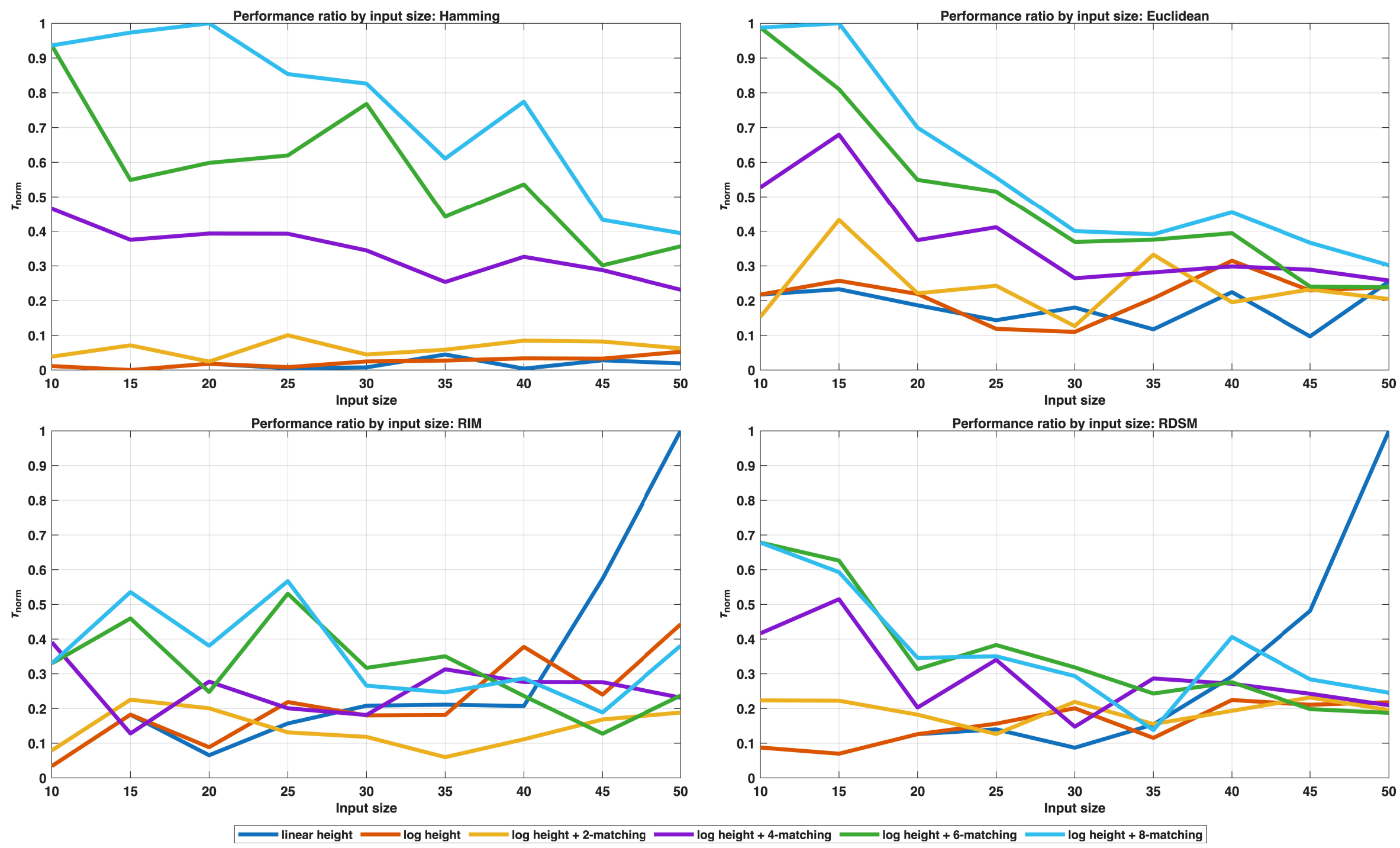

Figure S3: Mean performance ratios of SDP relaxation and profile rounding under different algorithm settings. X-axis represents input sizes, and Y-axis represents normalized performance ratio values $\tau_{norm} = \frac{\tau - 1}{\tau_{\max} - 1}$, $1 \leq \tau \leq \tau_{\max}$.

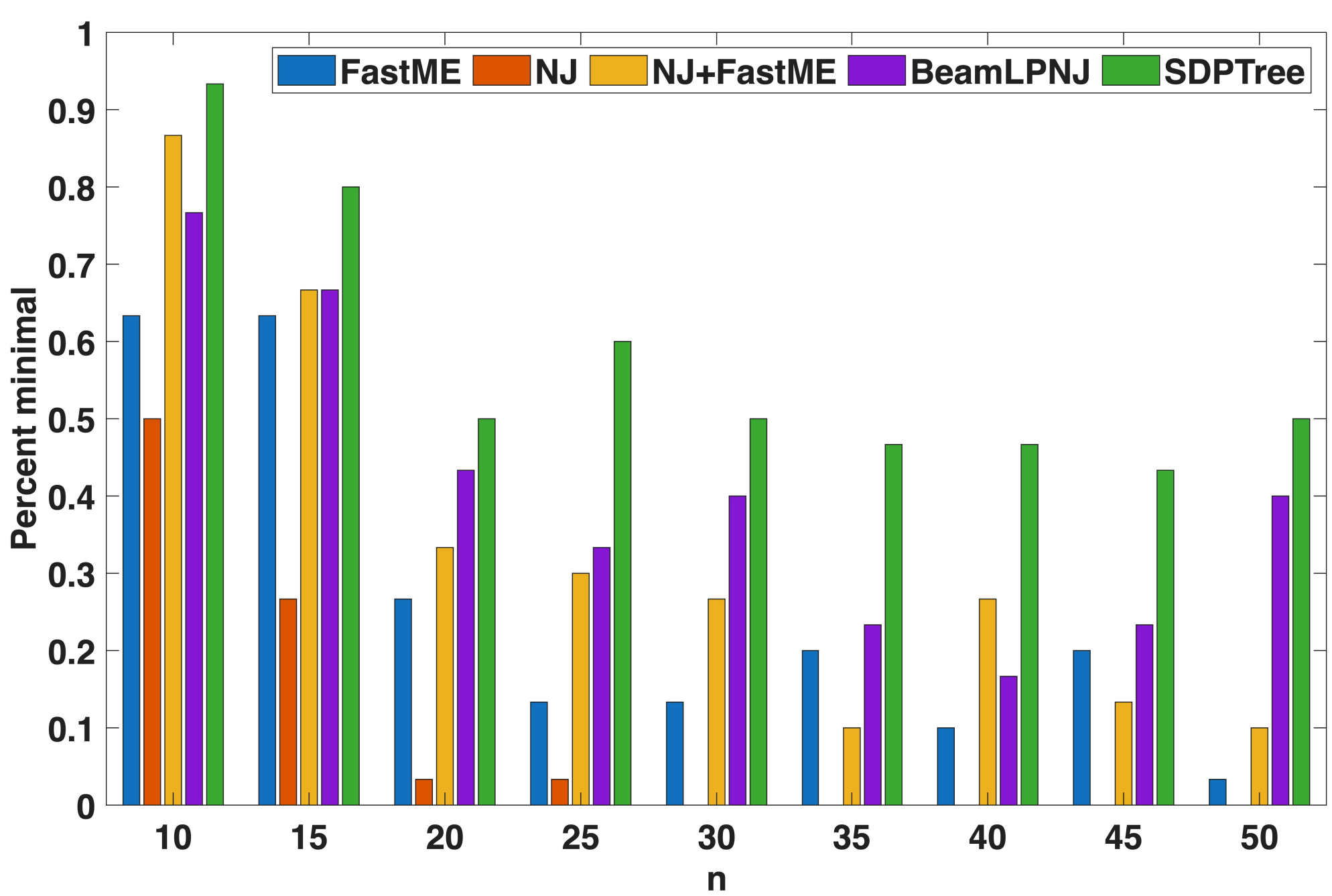

Figure S4: Joint comparison of four methods on simulated data (with the Hamming dataset excluded). The X-axis represent input size, and the Y-axis shows the percentage of instances where a method achieved the best objective value among the four methods (ties allowed).

	**FastME**	**NJ**	**NJ+FastME**	**BeamLPNJ**	**SDPTree**
**FastME**	0	0.711	0.333	0.328	0.175
**NJ**	0.267	0	0.056	0.075	0.050
**NJ+FastME**	0.525	0.867	0	0.45	0.225
**BeamLPNJ**	0.647	0.817	0.478	0	0.292
**SDPTree**	0.694	0.903	0.586	0.606	0

Table S1: Pairwise comparison of four methods on simulated data (with the Hamming dataset excluded). The number at the position (i, j) in the table is the percentage of test instances where the method i produces a better solution than the method j.

	**FastME**	**NJ**	**NJ+FastME**	**BeamLPNJ**	**SDPTree**
**FastME**	0	0.781	0.168	0.120	0.093
**NJ**	0.036	0	0.013	0.016	0.012
**NJ+FastME**	0.211	0.795	0	0.128	0.100
**BeamLPNJ**	0.242	0.799	0.209	0	0.114
**SDPTree**	0.263	0.803	0.228	0.167	0

Table S2: Pairwise comparison of four methods on Phylobench data. The number at the position (i, j) in the table is the percentage of test instances where the method i produces a better solution than the method j.

References

[1] Daniele Catanzaro, Martine Labbé, Raffaele Pesenti, and Juan-José Salazar-González. The balanced minimum evolution problem. *INFORMS Journal on Computing*, 24(2):276–294, 2012.

[2] Thomas M Cover. *Elements of information theory*. John Wiley & Sons, 1999.
